# Our Philadelphia Letter

**Published:** 1885-11

**Authors:** Harry Huzza

**Affiliations:** Philadelphia, Pa.


### OUR PHILADELPHIA LETTER.

OPENING OF THE REGULAR WINTER SESSION OF THE MEDICAL COLLEGES—PROFESSOR HOLLAND'S ADDRESS AT JEFFERSON—RESIGNATION OF PROFESSOR PANCOAST—THE CLINICS—THE FEMALE MEDICAL STUDENT AND HER MANNER OF EXAMINING PATHOLOGICAL SPECIMENS—THE NEW ANTISEPTIC, HYDRO-NAPTHAL—LARGE DOSES OF DIGITALIS, ETC.

*Editors Atlanta Medical and Surgical Journal:*

I wrote you last month rather early, just at the opening of the preliminary term. It takes a week or two to get everything in smooth working order—to initiate the new students into the ways of college life, and to give time for the professors and older students to become once more easy in harness. This preparatory period has its uses in another manner, it cools the fiery energy of the first-year men, who, fresh from home, are ready for anything. They would not hesitate to ligate the aorta, or amputate the head, and they skin a cadaver like a butcher would a beef. But in a week or so everybody settles down to steady work, and the introductory lecture opens the regular session most auspiciously.

This was delivered at Jefferson College by Professor Holland, his subject being "The Practical Value of Chemistry as Illustrated in the Life of the late Professor Rogers." The address was eloquent, scholarly and instructive. Professor Holland is the third teacher that Jeff. has received from Louisville, the former ones being the late Professor Gross and Professor Parvin.

By the way, Professor W. H. Pancoast has offered his resignation, to take effect at the end of the ensuing year. It is not known who will be his successor to the chair of anatomy. There are over four hundred and fifty matriculates at Jefferson, and about three hundred and fifty at the University of Pennsylvania. The senior classes are especially large.

No items of special interest in medicine have been sprung lately. The new antiseptic, hydro-napthal, proposed by Dr. Fowler, of Brooklyn, is being tried in the hospitals, but there has not been time for rendering a decision. It is said to be ten times as strong a germicide as carbolic acid, to have no odor or irritating qualities, and cannot be used in excess, as it is only soluble in water to a certain point. If experiment sustains these claims, it will prove valuable.

The clinics have been interesting, the more so as there have been quite a number of rare cases. In Professor DaCosta's clinic was presented a man with an aneurism of the innominate artery, in whom it was proposed to ligate the carotid and subclavian. A careful examination showed involvment of the aortic arch. The diagnosis, which was a very difficult one, turned on this point—that while the right pulse was strong, the left could not be perceived—in fact, had not been for twenty years, and the respiratory sounds on the right were very much exaggerated, showing pressure down in thorax. The treatment was iodide of potassium to point of tolerance, tr. aconite for heart thumping, and rest.

Strange to say, the same week there were two cases of the same kind at the Pennsylvania Hospital. One of them presented the added symptom of a persistent cough from nerve pressure, and irritation of the left vocal cord, which could only be relieved by a spray of cocaine. The *post mortem* specimens of a true, sacculated aneurism of the aorta were also shown. This clinic is largely attended by the students of the Women's Medical College. When this heart and aneurism were passed around, every one of the female students used a *hair-pin* to examine them with. It is needless to say there was much envy and laughter among the male M. D.'s, who had to use their fingers.

In connection with the next case, one of mitral heart dropsy, Dr. Meigs remarked that he had only lately learned how to produce the diuretic effect of digitalis ; that this was not widely known, and when he first tried it 'twas with fear and trembling. The method was to give the patient, say 5 drachms of tr. digitalis, to be taken, 30 drops=15 minims every four hours, day and night, but *stopped at once* if the amount of urine decreased, or the heart became feeble. This is practically 40 grs. of digitalis in three or four days, *beyond which it must not* be continued.

In the *Surgical Clinic*, at Jefferson, Professor Brinton performed perineal cystotomy on an old sailor for chronic vesical irritability and incontinence of urine. Incision was made through perineum in median line, bladder washed out and drainage tube inserted Treatment was milk and lemonade, with opium pills for pain. The patient did well, the tube being removed on the eighth day. He shaved on second day, and the Professor remarked that when a man shaved or a woman did up her back hair, it was a certain sign they would get well. Prof. Brinton also performed complete excision of the scapula for a rapidly growing sarcoma.

In the *Medical Clinic* I saw two typical cases that were both rare and instructive. The first was a true case of excessive appetite, boulimia. Patient, a man of twenty-four, required five large meals a day. If he abstained, it produced faintness, increasing to painful intensity. Professor DaCosta referred the disease to the nervous system, deranged functions of pneumogastric, most likely, in connection with trouble at the digestive centre of brain. The most certain remedy is fluoride of calcium, but its attendant nausea makes bromide of sodium far preferable when the disease will yield to it.

The second case was one of pure gastralgia, not from reflex causes, but of nervous origin, which is rather rare. The patient, a girl of nineteen, complained of excessive pain in epigastric region, fainted at times, suffered night and day, aggravated by exercise or excitement, and irrespective of food, in fact, more frequent on empty stomach, but no vomiting. The disease was pronounced very difficult to cure, the main thing being not to irritate the nervous susceptibility. Treatment was an absolute milk diet, allowing a soft-boiled egg if too irksome, arsenic internally and small doses of opium and cannabis indica for the pain.

Something of a curiosity was shown the Jeff. students recently by Professor Pancoast. The patient was a child five years old, sent from lower Pennsylvania, exhibiting total fœtal amputation of arms at elbows and legs at knees. He was remarkably bright, could hop around on the stumps of legs at a surprising rate, could scribble with a pencil and cut paper with scissors.

Professor Parvin also showed a unique specimen—a seven months premature child with its heart external to its body. The organ projected from the middle of the sternum, and there was no cavity or other malformation.

Speaking of curious things, two recent cases at Pennsylvania Hospital admirably illustrate that appearances are deceitful. The one was a man shot in upper part of right iliac region with a 32-calibre pistol by an assailant standing two yards in front of him. An examination showed that the ball passed in as far as the muscle, then turned at right angles downwards, and without injuring peritoneum, bladder or rectum, lodged in the perineum. A simple wound was the result.

The other was a man who was admitted to the hospital and died six days afterwards. When he entered there were diagnosed: 1st, an aortic regurgitant murmur; 2nd, mitral regurgitation; 3rd, emphysema; 4th, pulmonary apoplexy; 5th, was in third week of typhoid fever, with abdominal spots, peritonitis, etc. The *post mortem* showed that not only were these diagnoses correct, but he also had: 6th, abscess of the liver; 7th, eight gall stones; and 8th, an enlarged heart *totally adherent* to the pericardium. With that accumulation of disease, how did he live as long as he did?

But a due regard for your limits admonishes me to be brief.

Philadelphia has so much that is interesting, both medically and otherwise, that one's pen could scarcely tire of description. While she may be provincial, as New York declares, or, let us say, is less cosmopolitan, perhaps that very quality fits her for a medical center. Be that as it may, this is a fine city, and the points of interest, study and amusement are well-nigh innumerable. Perhaps I could not close better than with an abstract of a lecture on Pathology by Professor Formad, of the University of Pennsylvania. These are to some extent, perhaps, his own ideas, but they may be taken as the latest pathological research of those who oppose the recent discoveries of Koch. The main points were as follows :

All pathological processes may be resolved into five classes—hyperæmia, active and passive; thrombosis and embolism; inflammation; tumors; degeneration.

Passing by the first two, he defined the others as below:

Inflammation is the abnormal migration of white corpuscles.

Tumors are the aggregation, including proliferation, of cells of the body in one place, especially epithelial cells. The malignity of tumors is due to the migration or mobility of the tumor cell.

Degeneration is cell destruction.

Now, all diseases are either *acute* or *chronic*. Acute disease is always associated with desquamation and exudation.

Chronic disease is always associated with formation of connective tissue. Respectfully, HARRY HUZZA.

Philadelphia, Pa., October 15th, 1885.

